# Protecting nutrition in a food crisis

**DOI:** 10.2471/BLT.24.291393

**Published:** 2024-10-02

**Authors:** Anne Marie Thompson Thow

**Affiliations:** a Menzies Centre for Health Policy and Economics, University of Sydney, 1 City Road, Camperdown, 2006, Australia.

## Abstract

Food insecurity and malnutrition are rising worldwide due to disruptions in food systems related to interconnected health-, climate- and conflict-related crises. Although governments globally are committed to addressing nutritional challenges, policy responses have increasingly focused on food security and, particularly, on food affordability. However, these short-term measures often overlook the necessity of integrating nutritious foods into the food system to ensure improved long-term nutrition. By drawing on the United Nations Committee on World Food Security’s *Voluntary guidelines on food systems and* *nutrition*, this article outlines opportunities for policy-makers to integrate nutrition into key elements of the crisis response. Key policy areas where nutrition could be further integrated include social protection, agricultural investment, trade policy and urban planning. Strengthening the focus of nutrition in these measures will be essential to establish long-term incentives that support food systems transformation for improved nutrition. Drawing on theories of the policy process, I propose that stronger governance and cross-sectoral dialogue will be critical to achieve sustained nutritional outcomes. Health policy-makers can play a leadership role in supporting cross-sectoral policy change by carefully framing the policy issues, advocating for institutional structures that promote collaboration across sectors to prioritize nutrition, and strengthening the management of conflicts of interest in food system policy-making.

## Résumé

L’insécurité alimentaire et la malnutrition sont en augmentation dans le monde entier, en raison de perturbations des systèmes alimentaires liées aux crises interconnectées portant sur la santé, le climat et les conflits. Bien que les gouvernements du monde entier se soient engagés à relever les défis en matière de nutrition, les réponses politiques se sont concentrées de manière croissante sur la sécurité alimentaire et, en particulier, sur l’accessibilité financière aux aliments. Toutefois, ces mesures à court terme négligent souvent la nécessité d’intégrer des aliments nutritifs dans le système alimentaire afin de garantir une meilleure nutrition sur le long terme. En s’appuyant sur les *Directives volontaires sur les systèmes alimentaires et la nutrition* du Comité de la sécurité alimentaire mondiale des Nations unies, le présent article souligne les possibilités pour les décideurs politiques d’intégrer la nutrition dans les éléments clés de la réaction aux crises. Les domaines politiques clés dans lesquels la nutrition pourrait davantage s’intégrer comprennent la protection sociale, l’investissement agricole, la politique commerciale et la planification urbaine. Il sera essentiel de mettre davantage l’accent sur la nutrition dans ces mesures afin d’établir des mesures incitatives à long terme qui soutiennent la transformation des systèmes alimentaires en vue d’améliorer la nutrition. En m’appuyant sur les théories du processus politique, je suggère qu’une gouvernance plus forte et un dialogue intersectoriel sont essentiels pour obtenir des résultats durables en matière de nutrition. Les décideurs politiques en matière de santé peuvent jouer un rôle de premier plan dans le soutien au changement de politique intersectorielle en définissant soigneusement les questions politiques, en plaidant pour des structures institutionnelles qui favorisent la collaboration entre les secteurs afin d’accorder la priorité à la nutrition, ainsi qu’en renforçant la gestion des conflits d’intérêts dans l’élaboration des politiques relatives aux systèmes alimentaires.

## Resumen

La inseguridad alimentaria y la malnutrición van en aumento en todo el mundo debido a las alteraciones de los sistemas alimentarios causadas por crisis relacionadas con la salud, el clima y los conflictos. Aunque los gobiernos de todo el mundo se han comprometido a abordar los desafíos nutricionales, las respuestas políticas se han centrado cada vez más en la seguridad alimentaria y, en particular, en la asequibilidad de los alimentos. Sin embargo, estas medidas a corto plazo suelen pasar por alto la necesidad de integrar alimentos nutritivos en el sistema alimentario para garantizar una mejor nutrición a largo plazo. A partir de las *Directrices voluntarias sobre los sistemas alimentarios y la nutrición* del Comité de Seguridad Alimentaria Mundial de las Naciones Unidas, en este artículo, se esbozan las oportunidades que tienen los responsables de formular políticas para integrar la nutrición en los elementos clave de la respuesta a las crisis. Entre los ámbitos políticos clave en los que la nutrición podría integrarse más, se incluyen la protección social, la inversión agrícola, la política comercial y la planificación urbana. Reforzar el enfoque de la nutrición en estas medidas será esencial a fin de establecer incentivos a largo plazo que apoyen la transformación de los sistemas alimentarios para mejorar la nutrición. En base a las teorías del proceso político, se propone que una gobernanza más sólida y un diálogo intersectorial serán fundamentales para lograr resultados sostenidos en materia de nutrición. Los responsables de formular las políticas sanitarias pueden desempeñar una función de liderazgo en el apoyo al cambio político intersectorial mediante una cuidadosa formulación de las cuestiones políticas, la defensa de estructuras institucionales que promuevan la colaboración entre sectores para dar prioridad a la nutrición y el refuerzo de la gestión de los conflictos de intereses en la formulación de las políticas del sistema alimentario.

## ملخص

يزداد انعدام الأمن الغذائي وسوء التغذية حول العالم بسبب الاضطرابات في أنظمة الغذاء المرتبطة بالأزمات المتشابكة والمرتبطة بالصحة والمناخ والصراع. وعلى الرغم من التزام الحكومات على مستوى العالم بمجابهة التحديات الغذائية، فقد ركزت الاستجابات السياسية بشكل متزايد على الأمن الغذائي، وخاصة القدرة على تحمل تكاليف الغذاء. ومع ذلك، فإن هذه الإجراءات قصيرة الأجل غالبًا ما تتجاهل ضرورة دمج الأطعمة المغذية في نظام الغذاء لضمان تحسين التغذية على المدى البعيد. من خلال الاعتماد على المبادئ التوجيهية الطوعية لأنظمة الغذاء والتغذية التابعة للجنة الأمم المتحدة للأمن الغذائي العالمي، فإن هذه المقالة تحدد الفرص المتاحة لصناع السياسات نحو دمج التغذية في العناصر الرئيسية للاستجابة للأزمة. إن مجالات السياسة الرئيسية التي يمكن دمج التغذية فيها بشكل أكبر، تشمل الحماية الاجتماعية، والاستثمار الزراعي، والسياسة التجارية، والتخطيط الحضري. سيكون تعزيز التركيز على التغذية في هذه الإجراءات ضروريًا لتأسيس حوافز طويلة الأجل تدعم تحول أنظمة الغذاء لتحسين التغذية. وبالاعتماد على نظريات العملية السياسية، فإني أقترح أن الحوكمة الأقوى والحوار بين القطاعات سيكونان حاسمين لتحقيق نتائج غذائية مستدامة. يمكن لصانعي السياسات الصحية أن يلعبوا دوراً قيادياً في دعم تغيير السياسات عبر القطاعات، وذلك من خلال التركيز على الجوانب السياسية بعناية، والدعوة إلى إنشاء هياكل مؤسسية ترتقي بالتعاون عبر القطاعات لمنح الأولوية للتغذية، وتعزيز إدارة تضارب المصالح في وضع سياسات نظام الغذاء.

## 摘要

在健康、气候和冲突相关危机的相互作用下，粮食体系中断，导致全球粮食不安全和营养不良现象逐渐加剧。尽管全球各国政府致力于应对营养挑战，但是政策应对措施的重心却逐渐向粮食安全（特别是粮食负担能力）方面转移。然而，这些短期措施往往忽略了将营养食品纳入粮食体系来确保改善长期营养状况的必要性。通过借鉴联合国世界粮食安全委员会的《粮食体系和营养自愿准则》，本文概述了政策制定者将营养纳入危机应对措施相关关键要素的机会。可进一步纳入营养问题的关键政策领域包括社会保护、农业投资、贸易政策和城市规划。在这些措施中强调注重营养对于制定支持粮食体系向改善营养方向发展的长期激励措施至关重要。根据政策过程相关理论，我建议加强治理和跨部门对话，此举对持续改善营养状况将发挥至关重要的作用。通过仔细研究政策问题，倡导构建促进跨部门合作以优先考虑营养问题的体制结构，以及加强管理粮食体系政策制定中出现的利益冲突，卫生政策制定者可以在支持跨部门政策变革方面发挥领导作用。

## Резюме

Во всем мире растет число случаев нехватки продовольствия и недоедания из-за сбоев в продовольственных системах, вызванных взаимосвязанными кризисами, которые относятся к болезням, климатическим изменениям и конфликтам. Несмотря на то что органы государственной власти во всем мире стремятся решить связанные с питанием проблемы, политические меры реагирования все больше концентрируются на обеспечении продовольственной безопасности и, в частности, на доступности продовольствия. Однако при реализации этих краткосрочных мер часто упускается из виду необходимость интеграции полезных продуктов питания в продовольственную систему для обеспечения улучшения качества питания в долгосрочной перспективе. Опираясь на* Добровольные руководящие принципы по продовольственным системам и питанию* Комитета ООН по всемирной продовольственной безопасности, в данной статье авторы приводят описание возможностей для разработчиков политики по интеграции питания в ключевые элементы антикризисных мер. Основные области политики, в которых возможна дальнейшая интеграция питания, включают социальную защиту, инвестиции в сельское хозяйство, торговую политику и городское планирование. Усиление внимания к питанию в этих мероприятиях будет иметь важное значение для создания долгосрочных стимулов, способствующих преобразованию продовольственных систем в целях улучшения качества питания. Опираясь на теорию процесса выбора политики, авторы предполагают, что для достижения устойчивых результатов в области питания решающее значение будут иметь более эффективное управление и межсекторальный диалог. Разработчики политики в области здравоохранения могут играть ведущую роль в поддержке межсекторальных изменений в политике, тщательно формулируя вопросы политики, выступая за создание институциональных структур, способствующих сотрудничеству между секторами в целях определения приоритетов питания, и укрепляя управление конфликтами интересов при разработке политики в отношении продовольственной системы.

## Introduction

Malnutrition in all its forms is on the rise, and this is hampering the achievement of the sustainable development goals.^1^ The prevalence of undernourishment rose sharply in 2020 and has remained elevated at approximately 9% of the global population until 2023, affecting around 750 million people.^2^ In 2022, 69% (1.2 billion) of women of reproductive age (15–49 years) globally were deficient in at least one of three critical micronutrients (iron, zinc and folate).^3^ At the same time, obesity is increasing, with 881 million adults (16%) affected in 2022.^2^ This multiple burden of malnutrition is also evident among children. Of the estimated 663 million children younger than 5 years worldwide in 2022, an estimated 148 million (22%) were stunted, 45 million (7%) were wasted and 37 million (6%) were overweight.^2^

This rise in malnutrition is largely driven by disruptions to global food systems due to the coronavirus disease 2019 (COVID-19) pandemic, climatic events and regional conflicts.^2^^,^^4^ These events have had interconnected and changing impacts on food production, distribution and access, including reducing availability of inputs for agricultural production, decreasing crop yields, as well as disrupting transport logistics and the livelihoods of food systems workers and others.^2^^,^^4^^,^^5^ These impacts have contributed to a global food crisis, by contributing to income and livelihood collapse, food system shocks and uneven food price trends.^4^^,^^5^ As a result, healthy diets are unaffordable to more than a third of the global population, due to both high costs of nutritious foods and widespread poverty.^2^^,^^6^ This food crisis has also created economic challenges, such as inflation, cost-of-living increases, and reduced food affordability due to lower incomes,^7^^,^^8^ further exacerbating inequalities in nutritional status. These inequalities intersect across factors such as gender, education, economic and social status, location and ethnicity. Vulnerable populations have been disproportionately affected, including women, farmworkers and migrants, Indigenous populations, informal workers and people with disabilities.^9^

In response, governments worldwide are acting urgently to mitigate the effects of this crisis. Global commitments have been made to scale up the crisis response, noting the importance of redressing immediate effects on food insecurity and nutrition, together with increased attention to the affordability of food and strategic investment in food production.^7^^,^^10^ A core concern for food policy-makers is the economic dimensions of the crisis, including affordability and access to food.^5^ Key elements of the recommended policy response are thus economically focused, targeting income, food prices and food access. The policy responses include reorienting agricultural investment, expanding social protection policies, strengthening urban food systems and facilitating trade.^4^^,^^11^ Although governments worldwide remain committed to addressing the nutritional challenges, short-term responses have primarily focused on affordability and access, with limited consideration of nutritious foods that provide beneficial nutrients such as vitamins, minerals and other essential compounds, while minimizing the presence of potentially harmful substances.^12^

However, even short-term investments in food systems can create lasting legacies, influencing ongoing policy approaches and reinforcing connections between food system actors.^13^ The current challenge and opportunity for health policy-makers is to support immediate responses to the food crisis, while contributing to long-term food systems transformation for improved nutrition in the coming decades. This transformation can be achieved through integrating nutrition considerations into policy design. Embedding nutrition during periods of policy change can thus have lasting positive benefits, and avert potential negative outcomes in the long term, because malnutrition in all its forms comes with substantial social and economic costs, including direct health-care costs and lost productivity.^14^

Member States of the United Nations (UN) have committed to ensuring that immediate responses to the food crisis support, rather than undermine, long-term policy goals for food systems and nutrition.^4^^,^^7^ However, translating this commitment into action, that is, the integration of nutrition into policy measures related to agriculture, trade, social protection and urban planning, requires that policy-makers engage across multiple governmental sectors.^2^^,^^15^ In doing so, policy-makers must navigate and manage competing policy priorities and diverse stakeholder interests, including industry influence.^16^^–^^19^ Policy-makers also face underlying challenges to policy coherence for nutrition in the form of limited governance mechanisms and low capacities to support cross-sectoral engagement.^16^

As the health sector typically holds primary responsibility for nutrition, but the key crisis response measures lie within the mandate of other food-related sectors, this article explores how health sector policy-makers can contribute to integrating nutrition into a food crisis response. In addition, this article draws on political science theories to identify strategies for effective cross-sectoral engagement that will enable this integration.^20^^–^^22^ With strategic leadership and engagement of the health sector, this crisis could provide an opportunity for extending cross-sectoral dialogue on transforming food systems for improved nutrition.

## Integrating nutrition

Substantial efforts are needed to promote policy coherence on nutrition and food systems. This article addresses one important and timely question for health policy-makers: how can they support integration of nutrition in responses to the food crisis in a way that fosters long-term multisectoral progress on nutrition? This article draws on the *Voluntary guidelines on food systems and nutrition* to identify opportunities to strengthen integration of nutrition into short-term food systems measures, thereby improving long-term nutrition outcomes. Endorsed by the UN Committee on World Food Security in 2021, these guidelines identify evidence-based policy interventions aimed at reducing all forms of malnutrition through the promotion of sustainable food systems.^23^ They provide policy-makers and other actors with insights into a comprehensive approach that spans transparent governance; sustainable food supply chains; equal access to healthy diets; food safety; nutrition knowledge, education and information; gender equality; and resilient food systems in humanitarian contexts.

There are five key opportunities to integrate long-term nutritional considerations into the prioritized short-term measures which address the current crisis. Four of these opportunities focus on the key recommended actions for addressing food crises described above, including expanding social protection policies, reorienting agricultural investment, trade facilitation and strengthening urban food systems.^4^^,^^11^^,^^24^ The fifth is to strengthen monitoring and reporting across all these areas. These opportunities are based on the complementarities between food system and nutrition policy goals; these complementarities provide a foundation for integrating nutrition consideration into broader food system policies.^25^ In particular, improved nutrition contributes to, and benefits from, the achievement of shared whole-of-government policy priorities related to poverty reduction, human well-being, sustainable development (including economic productivity) and equity.^26^^,^^27^ These shared and complementary policy priorities can provide a platform for health sector engagement with food system policy-makers regarding nutrition. Specifically, health sector competencies in nutrition can inform the selection of diverse foods that contribute to healthy diets; provide information about nutrition and healthy diets; and ensure that equity and equality are considered in food systems policies. These opportunities reflect emerging evidence from practice and build on government efforts to date, and each opportunity is described in the following paragraphs, and supported with an example of integration into the policy area, drawn from global experience.

First, promoting consideration of nutrition in social protection and public procurement programmes offers an opportunity for long-term nutritional benefits by increasing consumer awareness of and access to nutritious foods. Integrating nutrition is aided by synergies with broader policy priorities focused on gender-sensitive measures and sustainable local food systems, particularly where nutritious local and/or traditional foods can be integrated into such programmes. Nutrition-sensitive social protection can improve nutritional outcomes, particularly for women and children.^28^ For example, transfer programmes targeted to households with children younger than 5 years, or transfers combined with communication interventions for nutrition behaviour change. Additionally, local food procurement can support livelihoods and food security for food producers and workers.^29^^,^^30^ The health sector can contribute to policy coherence between social welfare investments and nutrition by translating national dietary guidelines into specific guidance for school food and community-based meal programmes. For example, in Brazil, the implementation of criteria for purchasing school food from local family farms led to increased quantity and variety of healthy foods in schools in Santa Catarina.^31^ Health sector experts can also provide nutritional expertise and competencies to support behaviour change communications and the selection of supplementary foods.^32^

Second, integrating nutritional considerations into new agricultural investment can support long-term nutritional benefits by increasing production of nutritious foods, benefiting affordability and availability. Integrating nutrition is supported by potential synergies with agricultural priorities that promote traditional and local foods, as well as crop diversification and biodiversity, which improve dietary quality and diversity.^4^ For example, scaling up extension support and subsidies that target production and storage of traditional nutritious foods. The impact of the crisis on food affordability, together with changes in production inputs, namely reduced access to fertilizer, has led to renewed commitment to agricultural investment by governments. Important investments in resilience of agricultural production systems are being made, in response to both food systems disruptions arising from the COVID-19 pandemic and growing understanding of the impacts of climate change on agriculture.^33^^,^^34^ The health sector has the opportunity to support the integration of nutritional consideration into agriculture investments to promote increased availability of nutritious foods. For example, investments to improve access to agricultural inputs for foods such as poultry and vegetables, in conjunction with provision of nutrition information have been associated with increased production and consumption in the Plurinational State of Bolivia, Cambodia, India, Kenya and Nepal.^35^

Third, health policy-makers have the opportunity to advocate for inclusion of nutrition in food-related trade policy responses to the crisis, ensuring that any long-term policy changes proactively support access to nutritious food. The focus of responses to date has been on limiting trade restrictive measures and encouraging trade in accordance with a universal, rules-based, open, non-discriminatory and equitable, multilateral trading system under the World Trade Organization (WTO).^8^^,^^24^ This focus is crucial; however, an opportunity exists to use trade policy measures to promote diverse nutritious food and not simply staple crops.^36^ For example, the health sector can inform policies that promote trade in nutritious foods, such as neglected and underutilized crops rich in nutrients, while also advocating for reduced tariffs on healthy foods like fruit and vegetables. This approach has been used by the Cook Islands, Fiji, French Polynesia and Nauru to improve affordability of healthy foods.^37^

Fourth, there is an opportunity for health sector collaboration with urban planning and related policy sectors to support efforts to enhance food access in urban areas, particularly by promoting the integration of nutritious food access into long-term urban planning. Cities, and the ways in which they are linked to surrounding rural areas, are a critical focus for resilient food systems.^38^^,^^39^ In a crisis context, strengthening urban–rural linkages has been identified as an important area for policy attention to improve nutrition.^11^ For example, urban planning that enables access to fresh nutritious food through access to markets and land (e.g. urban and peri-urban farms), as well as through investments in food distribution and storage infrastructure.^39^ The health sector can complement this urban food agenda by integrating urban and peri-urban agriculture and land use into nutrition development strategies. This intentional complementarity between nutrition, urban planning and agriculture policy can, in turn, create synergies where renewed attention to urban food access during the crisis leads to improved access to nutritious foods for urban populations, particularly in low-income areas.^40^^,^^41^ Specific strategies include engaging with efforts to foster rural–urban linkages and with initiatives to develop innovations, resource hubs and new technologies in the food sector to support the availability of nutritious food. For example, in Nigeria, a recent analysis identified implicit consideration of nutrition at multiple points in urban planning documents at the national and municipal level, including support for microenterprises in the national housing policy and provision of decent markets in metropolitan development policies.^42^

In addition, ongoing monitoring and reporting on the nutrition impacts of the crisis will be critical in identifying effective future interventions and informing ongoing policy improvements.^43^^,^^44^ The health sector has essential capacities in food security and nutrition assessments and analyses, playing a key role in developing transparent mechanisms to monitor and evaluate (i) the nutrition impacts of both food system shocks and food systems policies, including equity dimensions; and (ii) the broader effects on food system stakeholders, including the distribution of transition costs and the cost and benefits of policy actions across sectors and actors.

## Health sector engagement 

Underlying all of these opportunities for the health sector to support inclusion of nutrition into specific policy measures related to the current food crisis is the broader question of how to foster policy convergence across sectors amid diverse interests and pressure of the growing food crisis. Such issues of governance are unlikely to lie within the remit of the health sector. However, health policy practitioners can take three actions to improve governance, informed by the *Voluntary guidelines on food systems and nutrition* and theories of policy processes.^45^^,^^46^

Political science points repeatedly to the power of framing and narrative in shaping policy.^20^ Gaining attention to nutrition as a critical complement to the crisis response will require a clear narrative, highlighting the importance of continued focus on nutrition and the potential long-term benefits created by short-term crisis response measures. This narrative must explicitly highlight the long-term benefits of integrating nutrition into these policy measures, the synergies and complementarities between improved nutrition and sectoral policy priorities, and how perceived trade-offs can be minimized. This narrative must also be embedded within a food systems framework that emphasizes sustainability, equity and human rights.^47^ Complementarities and synergies are also key to this narrative because they focus the dialogue on specific measures that achieve multiple objectives, including those related to human health, as well as environmental sustainability and economic livelihoods. Attention to nutrition as one of the key food systems outcomes is supported by growing evidence for the critical role of nutrition in ensuring that populations achieve their full economic and social potential.^48^ As such, adopting a long-term perspective to support nutrition does not undermine short-term responses, rather, it can support them. The World Bank’s Human Capital Project clearly demonstrates the potential for integration of nutrition to enhance the primary objective of short-term and economically focused crisis response measures. This synergy between nutrition and other policy objectives underpins the importance of designing short-term measures such that they do not inadvertently undermine achievement of improved long-term nutrition.^49^

Institutional structures are also critical in shaping policy outcomes.^21^^,^^22^ Health policy-makers can support transparent, inclusive and participatory governance processes for food policy, to enable integration of long-term food systems and nutrition priorities into short-term crisis response measures. The competencies needed to achieve effective crisis response measures lie across multiple sectors, including health, agriculture, social welfare, trade and economy. However, a persistent challenge for food and nutrition policy is the coordination and communication among the various sectors, forums, jurisdictions and actors with food policy interests, which is essential to promote policy coherence. Given that the mandate for leading the food crisis responses is likely to fall within the economic and social welfare sectors, the participation of the health sector in multisectoral governance forums will be necessary to ensure that nutrition is considered in food crisis responses. This participation will only be achieved if institutional structures are in place to support multisectoral governance involving all relevant actors. Where these structures do not yet exist, the health sector can play an important role in advocating for the creation of enabling environments for inclusive governance mechanisms. For example, supporting food and nutrition dialogues with indigenous populations, local communities and farmers to build shared knowledge, experience and insights. These efforts can be strengthened through global support for the integration of nutrition into food crisis responses which builds on initiatives for policy coordination to date across UN agencies, including the World Health Organization (WHO), Committee on World Food Security, Food and Agriculture Organization and United Nations Children’s Fund, as well as the World Bank, WTO and bilateral funding agencies. For example, scaling up coordinated actions and providing strategic support to Member States can facilitate learning from experiences, which can include successful cross-sector policy engagement and strategies for overcoming challenges.

Finally, the reality of increasing multistakeholder engagement in food system policy-making that brings together both the public and private sector presents an opportunity for the health sector to play a critical role in raising awareness about the need to manage conflicts of interest related to achieving nutrition policy priorities. The health sector is increasingly aware of the need for identifying and managing conflicts of interest to ensure policy outcomes that benefit the public interest.^50^ Identifying and managing potential conflicts of interest proactively can further promote sustainability of nutrition integration in non-health policy spaces and contribute to overcoming challenges. Drawing on the WHO Framework of Engagement with Non-State Actors and other initiatives, food system policy can be strengthened by increasing transparency and aligning with good regulatory practices. Additionally, developing codes of practice and institutional mechanisms is essential to ensure that conflicts of interests are identified and managed in cross-sectoral policy initiatives.

## Conclusion

Rates of food insecurity and malnutrition are rising globally, with food system shocks affecting supply chains and livelihoods in ways that have considerably affected food affordability. However, the change in context also represents an opportunity to strengthen policy in a way that transforms food systems for nutrition in the long term. To make the most of this opportunity for change, sectoral approaches to food system and nutrition policy must be better coordinated and engaged. Global and national policy-makers across sectors such as agriculture, social welfare, urban planning and trade play critical roles in leading policy responses. However, the health sector at both global and national levels can also assume a unique and essential leadership role in maintaining attention to nutrition across policy sectors during this crisis, and supporting improved governance and policy coherence. As the focus on food affordability and agricultural investment intensifies, responding to demands from consumers and producers to redress the immediate impacts of the crisis, this leadership will be critical to ensuring that the long-term incentives created from these short-term measures contribute to building better food systems.
